# The development of a health-promoting employment intervention with physical activity for young people Not in Education, Employment or Training (NEET): NEXT STEP—on the path to education and job

**DOI:** 10.1186/s40814-022-01174-1

**Published:** 2022-10-11

**Authors:** Teresa Victoria Høy, Andreas Jørgensen, Susan Andersen, Christina Bjørk Petersen, Maja Bæksgaard Jørgensen, Morten Grønbæk, Sengül Sarí, Laila Ottesen, Gro Inge Lemcke Hansen, Teresa Holmberg

**Affiliations:** 1 National Institute of Public Health, University of Southern Denmark, Odense, Denmark; 2 Danish Health Authority, Copenhagen, Denmark; 3 Centre for Team Sport and Health, Department of Nutrition, Exercise and Sport, University of Copenhagen, Copenhagen, Denmark; 4 The administrative department of Education & Students, University of Copenhagen, Copenhagen, Denmark

**Keywords:** Intervention development, Unemployment, NEET, Field study, Physical activity, Employment intervention

## Abstract

**Background:**

In the EU, approx. 16.5% of the 20–34-year-olds are Not in Education, Employment or Training (NEET). Research shows that good health is an important progression factor towards employment, and daily physical activity as well as participation in sports has a positive effect on physical and mental health as well as in the development of life skill. However, there is an absence of studies investigating what role physical activity can play in the employment efforts. The aim of this study is to investigate experiences with physical activity in employment interventions and to describe the development of a health-promoting employment intervention with physical activity for young people aged 18–30 years who are NEET.

**Methods:**

The study combined an evidence-based approach with practice-based experiences. It included a Mapping of Danish employment interventions with physical activity for young people NEET, an overview of national reports and international literature, a workshop with leaders and employees from Danish municipalities, and fieldwork in four municipalities. Key findings were grouped thematically and were transformed into intervention content in a close dialog with relevant researchers and practitioners.

**Results:**

In general, physical activity was assessed as a meaningful method when working with young people NEET. A range of positive experiences was highlighted, including successful experiences, surplus of mental resources, new ways of meeting, and new social relations. Based on these findings, a 16-week health-promoting employment intervention, NEXT STEP, was developed with the following intervention components: (1) fixed boundaries, (2) body and physical activity, (3) social relations and network, and (4) life skills and future. NEXT STEP is designed to be implemented in Danish job centers.

**Conclusion:**

The study found the great potential of including physical activity in employment interventions for young people NEET. The planning and development of the intervention have reinforced a comprehensive evaluation; however, the first intervention in its entirety is ready for testing in a randomized feasibility trial.

**Supplementary Information:**

The online version contains supplementary material available at 10.1186/s40814-022-01174-1.

## Key messages regarding feasibility

1. What uncertainties existed regarding the feasibility? Knowledge on how to combine the health and employment effort for young people (Not in Education, Employment or Training NEET) is lacking. This also applies to experiences of including physical activity in employment interventions among young people NEET and employees in Danish job centers.
2. What are the key feasibility findings? The study found the great potential of including physical activity in employment interventions for young people NEET. However, young people NEET is a very diverse target group, some of them struggling with a range of mental, physical, and social challenges. This degree of complexity demands complex and holistic interventions helping young people NEET towards employment or education.
3. What are the implications of the feasibility findings for the design of the main study? The intervention will cover a full employment intervention at the job center and not as an add-on offer as initially planned. Moreover, all activities must be carefully planned and executed by a competent and empathetic instructor taking the full range of physical, mental, and social barriers among young people NEET into account.

## Background

In the EU, approx. 16.5% of the 20–34-year-olds are Not in Education, Employment or Training (NEET) [1]. The NEET term is used as an overall category covering a heterogeneous population including the unemployed and school dropouts. The unemployed are only a subgroup of the broader category of NEET. During the COVID-19 pandemic, there has been a rise in the proportion of young people being NEET, also when looking at the specific numbers of unemployed [2].

In Denmark, it has been estimated that young people who never enter employment account for DKK 105 billion in expenses and lost income per youth year group measured over a life course [3]. Long-term unemployment at a young age has a direct effect on health and makes the chances of being employed later in life less likely [4]. Many young people NEET, especially those being long-term unemployed and without education, are vulnerable in terms of poor physical and mental health and social exclusion that may keep them in a marginalized situation that could persist into adulthood [5–8]. This group may be so far away from entering educational activities or employment, and therefore, employment interventions aiming at increasing labor market readiness or progression towards employment or education are a more realistic short-term goal rather than aiming for employment or education as a first step [9, 10].

Unemployment can adversely affect self-perception and self-esteem, and it is well-known that employment and health are connected [11]. The immediate health effects are particularly pronounced for young people. For example, the increased likelihood of depression as a result of unemployment is amplified for unemployed young people [4], and the prevalence of smoking, unhealthy dietary habits, and physical inactivity is significantly higher among unemployed [12] and among young people NEET in general [13, 14].

The individual mental and physical state of health can affect the ability to complete an education as well as to obtain and keep a job. A good state of health, both mental and physical, is one of the most important progression factors on the path to employment [9, 10]. Poor self-rated health negatively influences job search cognitions and coping resources, which in turn results in less active job search behavior and lower likelihood of employment [15].

It is well-documented that daily physical activity can contribute to more energy, successful experiences, increased well-being, and higher self-esteem and better self-rated health [16, 17]. Studies have shown that interventions that include physical activity generally lead to better mental health among unemployed in the adult population [18] and less anxiety and fewer symptoms of depression [19]. Moreover, it has been shown that physical activity as well as participation in sports can help to increase life skills (e.g., cognitive and social skills) among socially vulnerable young persons [20]. Studies have also directly linked higher levels of physical activity with positive labor market outcomes including reemployment [21, 22] as well as learning outcomes [23, 24] and higher levels of education [25, 26]. Four mechanisms have been proposed to explain why physical activity and sport participation leads to these positive labor market outcomes: (1) health, (2) network, (3) cognitive and non-cognitive skills, and (4) signaling [27]. All in all, this emphasizes the potentials of promoting physical activity and health among young people both unemployed and the group of NEET overall. However, there is an absence of studies investigating what role physical activity can play in the employment effort as interventions with physical activity have only been tested in very few studies with education and work as well as work readiness as outcomes [28, 29].

The aim of this study is to investigate experiences (e.g., acceptability) of including physical activity in employment interventions among young people NEET and employees in Danish job centers in order to develop an intervention to be tested in a later randomized feasibility trial. This study describes the rationale and methods used for developing the health-promoting employment intervention with a specific focus on physical activity for young people aged 18–30 years who are NEET in a Danish context with the overall goal to promote health and increase the enrolment in employment or education.

## Methods

### Setting and target group

The study was undertaken in the context of the Danish job centers for the target group of young people NEET. There are 94 public job centers in Denmark, corresponding to approx. one in all of Denmark’s 98 municipalities [30] (some job centers are shared). According to *the Consolidation Act on municipal action for young people under the age of 25* from 2017, all municipalities are obligated to offer a cohesive and coordinated youth effort across social, educational, and employment administrations [31, 32]. However, the organizational structures, health promotion policies, and specific activities in the programs differ [33, 34], e.g., by involving private providers more or less. Some municipalities have physically unified their efforts targeting young people NEET and establishing a youth center which coordinates the efforts across administrational offices.

Based on the Danish social security benefit reform from the Ministry of Employment in 2013 young people between the age of 18 and 30 who apply for social security are categorized into different groups depending on (1) whether they have a diploma from general or vocational upper secondary education and (2) the caseworkers’ assessment of the young person NEET’s readiness for education or employment [35]. Individuals with a diploma can be categorized as *ready for employment* or *ready for activity* (Table 1) [36]. For persons without a diploma, the main goal is education. They can be categorized as *clearly ready for education*, *ready for education,* and *ready for activity* as described below (Table 2) [37]. Individuals with or without a diploma who are *ready for the activity* will be offered additional interventions to help them improve their competences, clarify their needs, etc. [36–38].

**Table 1:** Overview of the categories for individuals under 30 years with a diploma

**Individuals under 30 years with a diploma from general or vocational upper secondary education**	***Ready for employment*** The individual can undertake employment and be self-sufficient within 3 months	***Ready for activity*** The individual has one or more challenges that need to be addressed before being ready for employment

**Table 2:** Overview of the categories for individuals under 30 without a diploma

**Individuals under 30 years without a diploma from general or vocational upper secondary education**	***Clearly ready for education*** The individual must start an education as soon as possible	***Ready for education*** The individual might need lessons in writing, reading or math before being able to begin and complete education on ordinary terms	***Ready for activity*** The individual has one or more challenges that need to be addressed before being ready for education

The study will include young people NEET between 18 and 30 years of age who are both *ready for activity* and *ready for education* but not those *ready for employment* or *clearly ready for education*.

### Design

The study took place over a nearly 2-year period (August 2016–June 2018) and included the following elements:

- Mapping of Danish municipality-based employment interventions with physical activity for young people NEET (August 2016-June 2017)
- Review of the international literature of employment interventions with physical activity among young people NEET (autumn 2017)
- Review of national reports on employment interventions among young people NEET (autumn 2017)
- Workshop with leaders and instructors from five municipalities (August 2017)
- Fieldwork in job centers in four municipalities (autumn 2017)

With these elements, we wanted to examine existing literature and practices to identify evidence and experiences working with young people NEET. We were interested in young people NEET as a target group in the Danish job centers and in the general methods used by the professionals. Additionally, we were interested in discovering how physical activity has been used in employment interventions, how young people NEET experienced being part of an employment intervention (including those with physical activity) as well as strengths and challenges for implementing NEXT STEP.

We did not define the methods for developing the intervention a priori. It was an iterative process that was guided, though not rigidly, by the intervention mapping method for planning health promotion interventions [39]. However, as an initial aim was to test the use of physical activity in an employment intervention, the method was not used in its full form. Moreover, as alignment with practice was very important in the intervention development process, we combined an evidence-based approach with practice-based experiences.

### Mapping of Danish municipality-based employment interventions with physical activity for young people NEET

From November 2016 until June 2017, we conducted a survey in the Danish job centers to map existing employment interventions with physical activity for young people NEET under the age of 30 in Denmark [40]. The survey was distributed to a relevant employee at each of the 94 job centers in Denmark. Seventy-five percent of the job centers responded to the survey.

The survey was developed in dialog with the Local Government Denmark (the association and interest organization of the 98 Danish municipalities) and was tested by three employees at Danish job centers and validated using cognitive interviewing [41]. The survey consisted of 58 questions regarding the number of interventions with physical activity for young people NEET, specific questions about the interventions (financing, content, evaluations, etc.), and questions regarding the employees’ views on the use of physical activity in employment interventions in general.

Furthermore, we conducted six qualitative and exploratory follow-up interviews with employees from six selected job centers who responded to the survey. The job centers were chosen based on positive and/or negative experiences with the use of physical activity for young people NEET. The follow-up interviews included themes about the organization, intervention content, barriers, and facilitators of implementation, recruitment, retention, and general challenges depending on the experiences of interest. The follow-up interviews were conducted by telephone and lasted approximately 30 min.

### Review of national reports on employment interventions among young people NEET

We did a literature search of existing Danish reports to gain information on the target group of young people NEET including descriptions of the target group and their challenges, recommendations for employment interventions, and evaluation of existing employment interventions. We used a snowballing method searching for gray literature through resources like The Danish Center for Social Science Research (VIVE) and the Danish Agency for Labor Market and Recruitment (STAR). A similar search was done on Google and Google Scholar using search terms like unemployment, labor market, young, efforts, intervention, and the similar terms in Danish.

### Review of the international literature on employment interventions with physical activity among young people NEET

We conducted a literature review to give an overview of the design and effect of employment interventions with physical activity for the group of young people NEET. For this purpose, the databases PsycINFO, PubMed, and Embase were used. First, relevant search terms were defined, and search blocks were created. The relevant terms to be used in the blocks were physical activity and unemployment, and similar terms were added to each block. A block specifically for young people was eliminated because of a lack of results. Finally, the term RCT and similar terms were added to the string aiming to specify the search (see Additional file 1: search string). We read the abstracts and excluded articles based on language, study design, or absence of physical activity in the intervention.

### Workshop with municipalities

A workshop (“Physical activity as a part of the employment intervention for young people NEET: workshop with municipalities, job centers and researchers”) was held in August 2017. Seven Danish municipalities were represented in addition to a representative from Danish People’s Aid, two researchers (THO and TEVH) from the National Institute of Public Health as well as two representatives from the Center for Health Promotion in Practice, Local Government Denmark who facilitated the day. The purpose was to share knowledge about the group of young people NEET and to discuss their experiences and the potentials and challenges using physical activity as part of an employment intervention in municipalities. We presented the findings from the Mapping of Danish municipality-based employment interventions with physical activity for young people NEET and the overall ideas of the project as an introduction to group-based discussions. The common characteristics and main conclusions from the discussions were written on a whiteboard in the room and noted on a computer for subsequent use in the development process.

### Fieldwork in municipalities

In September and October 2017, we conducted fieldwork in four municipalities (Table 3). The field visits (lasting between 3 and 6 h) included observations of specific courses for young people NEET, focus group interviews with young people NEET, and formal and informal talks with instructors^1^ and leaders and young people NEET, depending on the possibilities in each municipality. The planned focus group interviews were structured by an interview guide with themes and specific questions to guide the interview. Additionally, we engaged in conversations and informal interviews when the situation allowed it. Field notes from these conversations were written concurrently or as soon as possible. These notes captured what was said, what young people NEET participated in, how they participated, descriptions of the setting, the specific activities, the organization of the activities, etc. The notes were afterwards gathered, written into expanded texts, and subsequently, compiled into summaries of each field visit. The project group thematically grouped the summaries and findings from the field visits.

**Table 3:** Overview of the visited municipality-based employment interventions

	Young people NEET and instructors present	Setting	Purpose	Content	Methods
**A**	7–10 young people NEET *ready for activity* and one instructor	Municipality youth center	Four-week course in robustness and mental readiness	Presentations on stress and motivation, yoga and mindfulness, songwriting, etc.	Observation and focus group interview
**B**	10–15 young people NEET who are socially and mentally very challenged and two instructors	Facilities at a weightlifting club	A continuous offer with physical activities twice a week (for most individuals combined with other activities at the job center)	Circuit training and team sports	Observation, interview with the leader of the weightlifting club, and informal talks with young people NEET and instructors
**C**	7–10 young people NEET and one instructor	Youth center and adjacent buildings	Four-week screening course with the purpose of clarifying the participants’ current situation and the best suitable employment intervention	Visits to companies and local educational institutions, lectures in economics, job search, and social events including, e.g., physical activity	Observation, interviews with young people NEET, informal talk with leader and instructor
**D**	7–10 young men NEET with criminal or abusive backgrounds and two instructors	Fitness gym adjacent to a public swimming pool	13-week intervention with the purpose of preparing the young men NEET for education or employment, up to 25h/week	Combined individual training in a fitness center and group-based CrossFit sessions, vocational elements, communal eating, etc.	Observations and informal interviews with instructors and young people NEET
**E**	10–15 young people NEET and two instructors	Boxing club	13-week course with the purpose of preparing the young people NEET for education or employment, three times per week	Group-based intervention combining boxing and dialogs with a mentor. The young people NEET are offered a membership of the sports club	Observations and informal interviews with instructors and young people NEET

Overall, the fieldwork provided insight into the everyday life at a Danish job center, the motivation and barriers of young people NEET, the complex challenges seen from the perspective of the instructors and other professionals involved, and information about existing interventions with physical activity and inspiration to components of the NEXT STEP intervention.

All four visited municipalities (covering five different municipality-based employment interventions) used a combination of physical activity and other activities in the employment interventions, but with great variations. In two of the visited municipality-based employment interventions (A and C, Table 3), physical activity was a minor component of the intervention, and in the last three visited municipality-based employment interventions, physical activity was a major part of the municipality-based employment intervention (B, D, and E, Table 3).

### Method of development of the intervention components

The key findings from the explorative study investigating the experiences of including physical activity in employment interventions formed the basis for developing the intervention and its specific intervention components. The process of translating our explorative findings into specific intervention content took place in a dialog with the project partners, who all gave valuable feedback: instructors and leaders from Slagelse municipality, the Danish Outdoor Council, Preben Bertelsen (Aarhus University), Nye Stemmer ([New Voices], a company working with vulnerable groups), Danish People’s Aid, Center for Physical Activity Research (Copenhagen University Hospital), Center for Team Sport and Health (University of Copenhagen), Center for Health Promotion in Practice, and members of an Advisory board consisting of representatives with knowledge of intervention projects in various settings as well as employment interventions, physical activity, labor market, municipal organization, implementation, etc.

## Results

### Results from the mapping of Danish municipality-based employment interventions with physical activity for young people NEET

Our mapping of Danish municipality-based employment interventions with physical activity for young people NEET showed that 92% of the participating municipalities have had an employment intervention with physical activity for young people NEET in the past year or used to have one [40]. The employment interventions varied highly in terms of form, content, target group, and organization. In general, physical activity was most often included as a sub-element in the employment interventions for example by using the facilities at a local fitness center, which almost half of the employment interventions included. Also, the effects of the interventions were usually scarcely evaluated. Nevertheless, it was demonstrated that 86% of the participating municipalities assess physical activity as a meaningful method in the work with young people NEET. Three central themes emerged from the interviews with the municipalities in the mapping. Firstly, the nature and outdoors are useful settings, e.g., walking outside can function as an important setting for informal conversation in smaller groups [40]. Secondly, social relations and community are important both between the young people NEET but also between the job center employees and the young people NEET. Secondly, motivation and attendance can be enhanced through individual and common goals and mutual responsibility for each other, and smaller groups can give a feeling of exclusivity by creating a setting that is set apart from the ordinary procedures at the job center [40].

### Results from the reviewed literature

#### International literature

Regarding international literature, the search resulted in 136 articles at that point; however, after the screening process, only five studies were relevant. All studies were published between 2004 and 2015 and used physical activity as a method in employment interventions [18, 19, 28, 29, 42] (see Additional file 2: overview of the included articles). In general, there is very limited knowledge about interventions incorporating physical activity to promote physical and mental health among people outside the labor market [18, 42, 43], and we found none relating to young people NEET. Four of the studies include an adult population (30+ years) of long-term unemployed people [18, 19, 28, 42], often with poor health or health problems [28, 29, 42], and one study included 18–64-year-olds [29]. The results of these studies indicate that physical activity-promoting interventions can significantly improve the physical [19, 28, 42, 43] and mental health [18, 19] of the unemployed. However, employment interventions with physical activity have only been tested in very few studies with work and work readiness as outcomes [28, 29], but the results are promising. In a British study from 2009 using a non-controlled design including long-term unemployed people with back problems, almost 40% were employed and 23% enrolled in internships after a 6-week intervention with physical activity and individual counseling [29]. At that point, no controlled trials or effect studies targeting young people NEET have been conducted.

#### Danish national reports

We found 15 Danish reports relevant to include in the development process [9–11, 44–55]. The reports are published in the period from 2011 to 2019 by different organizations such as the Danish Committee for Health Education [9], The Danish Center for Social Science Research [10], and the Danish Agency for Labor Market and Recruitment [46]. Young people NEET (especially the group of young people NEET categorized as *ready for activity*) are often challenged on several mental, physical, and social parameters making them not immediately ready for education or employment [9, 48], and thus, there is a need for cross-sectional holistic interventions [11]. The literature also describes the importance of addressing individual challenges and barriers in the process of reaching a more desirable situation [1, 4, 10], and that some may have difficulties attending classroom-based lessons [45–47]. Moreover, the literature highlights the importance of safe, trustworthy, and authentic employees, who are willing to address individual challenges and help with practical issues in everyday life [44, 45, 49]. An evaluation from 2011 indicates that interventions with physical activity for young people NEET help create successful experiences in terms of pursuing goals, creating more structure, increasing daily coping skills, and increasing self-esteem and self-confidence [44]. Additionally, we were inspired by literature describing intervention methods and examples with positive outcomes for the group of young people NEET [44–46, 49, 50], e.g., that young people NEET with mental challenges can benefit from training in social situations to enhance social competences and that guidance in healthy lifestyles can contribute to increased energy and feelings of well-being [46].

Some reports focused on progression factors for obtaining a job or starting an education when measuring the effect of employment interventions [52–55]. The Employability Indicator Project initiated by the research center Væksthusets Forskningscenter^2^ in 2012 investigated the link between these progression factors for labor market readiness and the probability of future employment [9, 52, 53, 55]. Progression factors can be used to predict whether vulnerable recipients of social security benefits will start searching for a job, and whether they will obtain a job, and if these factors improve, there is a statistically significant probability that the individual will move closer towards education or employment [53–55]. Progression factors may be influenced by certain efforts and may develop over time [53]. Therefore, it is important to align the intervention and related outcomes with these progression factors.

The progression factors are [9, 53]:

A. *Self-efficacy* describes the belief in one’s abilities in general and in finding a job.
B. *Self-rated health* describes if and how many physical or mental challenges are a barrier to future employment or education.
C. *Job identity* describes if the individual has a clear goal about the near and distant future and if there is a meaningful plan going forwards to obtain a job.
D. *Social skills* describe individual competences in making and entering social relationships and the ability to cooperate with colleagues and fellow students.
E. *Social support* describes the experience of having social support from the closest network, such as families and friends and being a part of a community with like-minded people. Social support also includes the relationship with the caseworkers/instructors at the job center that ensures that the flow of information from the individual to the professional is smooth and thus that the right intervention is given at the right time.
F. *Caseworkers’ assessment* describes the professional’s assessment of the individual readiness for employment or education but also the caseworker’s/instructor’s expectations for the progression towards employment or education.

### Results from workshop and fieldwork

We present key findings from the exploratory study that also provided knowledge to develop the intervention and the specific intervention components.

#### Key finding: the need for structure and regularity in daily life

Our fieldwork indicated that many of the young people NEET lack an everyday life with structure and overview. If they are not obliged to arrive somewhere at a specific time, they will most often stay at home, which can lead to a daily life without routines, regular practical chores, and social events. According to instructors and the young people NEET themselves, it can be a challenge to get up in the morning, especially if the young people NEET must arrive at a new and unknown place. One instructor (case B) described it this way:

*“It’s a challenge for many [young people NEET] to show up here in the morning, but if they’re not here on time, they will have their social security benefit reduced. They must learn to be here on time because it’s demanded in the real world.”*

The young people generally expressed a desire for regular daily routines with fixed schedules and preferably also a fixed group of participants to avoid frustration, stress, and uncertainty caused by ongoing enrollment in employment interventions. Furthermore, findings indicate that the first few weeks are of great importance, and to assure a smooth transition process, it may be beneficial with initial information meetings where the young people NEET meet each other and the instructors and see the physical setting for the first time. A meeting like this should signal “I take care of you,” so the young people NEET feel comfortable and safer attending the employment intervention. Moreover, putting pressure on the young people NEET should be avoided in the first few weeks. “Ease us into it” as a young man NEET (case A) said and explained:

*“The first days are very vulnerable, because like ‘do I really want to be here?’ and ‘how is the vibe here?’. It’s difficult because it* [*the program of the day*] *cannot be too advanced on day one, but still people don’t want to sit here for six hours thinking ‘what am I doing here?’*

Additionally, it is beneficial to have common rules for participation, especially in the beginning, and to ensure a sense of security. For example, as highlighted by participants and instructors in case B: if someone does not actively participate in the activities “they should not point fingers at the others.” In case of injury or other reasons for not actively participating, that person could for example instead oversee timekeeping, etc.

#### Key finding: health promotion and physical activity—methods to motivate

A key observation throughout the workshop and fieldwork was that physical activity can help the young people NEET to become part of a community and to meet other young people in a similar situation. One young man NEET (case E) explained:

*“I have been on and off social security benefits since I was 18, and now I’m almost 28. This is the first offer that got me moving forward. The exercise makes me want to get up in the morning and gives me a reason to get up. Otherwise, I sleep until 1 p.m. and in the end don’t care anymore …”*

In general, instructors expressed a range of positive experiences using physical activity as a method when working with the target group. In addition to the immediate health effects, the instructors highlighted how physical activity creates successful experiences, surplus of mental resources, new ways of meeting, and new social relations (both between the young people and in the relation to the instructors).

Two interviews underlined that the young people NEET have ambitions of being physically active and that the level of physical challenge can be high. In one municipality, we observed how a group of the young people NEET were hard-working in a ballgame and in following circuit training. In another municipality, a woman NEET suggested activities like rappelling, and we asked if activities can be this challenging. The young woman NEET (case A) answered:

*“Yes, they can, but it must be a while into the employment intervention, so I know the people I’m doing the activity with. The setting will then be safe when the boundaries are pushed a bit.”*

Instructors highlighted the fine balance between the right amount of physical challenge and adequate consideration for individual challenges and barriers. Nevertheless, we observed that facilities for physical activity do not necessarily require a lot of space and a specific type of facilities.

In one municipality, the physical activity in the employment intervention took place in a sports club, and the young people NEET were offered an additional membership to the affiliated sports association. This enabled more opportunities for training as well as important experiences of being a part of a local sports association.

Despite the positive experiences with physical activities for the group of young people NEET, also challenges were observed. Firstly, a mutual concern expressed by the practitioners at the workshop was the implementation of an employment intervention including physical activity in the job centers taking the regular procedures into account. The importance of planning the intervention to cover a full employment intervention and not as an add-on offer with physical training as first presented and discussed. The argument was that the job centers do not have the resources to manage an additional employment intervention outside regular procedures. Moreover, attendance would probably be low if it were not mandatory.

Secondly, instructors also noted during the fieldwork that some of the young people NEET are not used to be physically active. They might have negative experiences exercising and may not know the feeling of being physically exhausted, including having sore muscles after a session of physical activity. They suggested to include an introduction of basic knowledge of how to ensure optimal sleep, nutrition, and body functioning—all factors important to address in an employment intervention with physical activity for this group of young people NEET. It must also be considered that the participants might not own sportswear, including clothing for outdoor exercise in cold weather.

#### Key finding: social relationship and the feeling of community are essential

Our observations and discussions with leaders and instructors showed that the feeling of community and positive social relationships are essential for the young people NEET. According to an instructor in one municipality, there is a general feeling of exclusion and disadvantage among the young people NEET, why it is important to include them in new and meaningful networks to gain confidence.

According to the young people NEET and the instructors, it was essential meeting other young people in a similar situation to avoid loneliness and to have something meaningful to wake up to; otherwise, the everyday life becomes unimportant and uninteresting. An instructor (case C) describes a situation where common and fun experiences together contribute to a feeling of community:

*“On Mondays, we [the two instructors in charge of the employment intervention] can feel if the group has done something fun together the week before. They have something to talk about.”*

Also, the relationship with the instructor is crucial: both in the short and long term. Authenticity and empathy are highly important as well when working with this target group. Likewise, the instructors must engage with the young people NEET but also be able to make demands. According to instructors, this could for example include commitment outside regular working hours as some of these young people NEET do not have others to ask for help.

In two municipalities, we observed that physical activity can have a positive impact on the relationship between the young person NEET and the instructors if the instructors participate in the activities. This involvement changes the hierarchy and context, contributing to a positive feeling of equality in an otherwise unequal relationship. Their participation also enables them to see and interact with the young people NEET in another way than usual; hence, they are given a better understanding of both strengths and weaknesses to guide the young people NEET into education or employment. As one instructor (case B) said:

*“We [the two instructors] try to participate in the activities because we get on the same level, and it creates trust. We get to talk with them [the young people NEET] in another way, and we can set ourselves apart from other authorities whom most of the young people NEET have a strained relationship to.”*

It was also mentioned as an advantage to include volunteers or positive role models into employment interventions for the young people NEET. However, it is important that they are well prepared for the task ahead but do not get involved in the specific cases of the young people NEET.

Finally, in two municipalities, we observed that meals are a useful method to create room for dialog. The experience was that meals eaten together create an informal talk across the table, and the meals are a social element most of the young people NEET miss in their daily life.

#### Key finding: the need for addressing multiple individual challenges

The leaders and instructors pointed to the need of handling individual challenges as the young people NEET are facing many challenges of physical, mental, or social character. One young man NEET (case A) had a quiet straightforward way of expressing this:

*“People are here because something is not working.”*

Therefore, the young people NEET have different needs and requests with regard to the content of the employment intervention, and instructors discuss the challenge of planning lessons suitable for everyone.

For example, employment interventions delivered in a classroom can cause unrest and anxiety due to previous bad experiences with school making some young people insecure about the general classroom setting. One young man NEET (case C) said:

*“School doesn’t work for me. I’m better at working. I like being active. I get so sleepy in a room like this and I can’t concentrate.”*

Another important finding was that the management of personal finances often is a challenge among the young people NEET. In general, it is an area of concern, highlighting a need for support to gain overview of their financial situation and find simple methods to manage it going forward. In one municipality, they used grocery shopping and shared preparation of meals as useful methods to help the young people NEET gain a better overview of their finances. An instructor (case C) said:

*“As part of the financial theme on Wednesdays, we sometimes plan a meal, go shopping for the groceries and cook the meal together. They really like it because it’s useful and enjoyable.”*

Instructors also told us that the job center demands a final evaluation of the individual progression towards employment or education after participation in an employment intervention. This is especially important for the young people NEET farthest away from employment or education (those in the category *ready for activity*) since every little progression may be a large step towards employment or education. The evaluation may include a description of the elements that the individual has been through and what knowledge and skills he or she has acquired during the period. Additionally, it must include a concrete plan for each participant with suggestions for relevant interventions henceforth.

### Program theory, intervention components, and discussion of potential outcomes

**Fig. 1:**
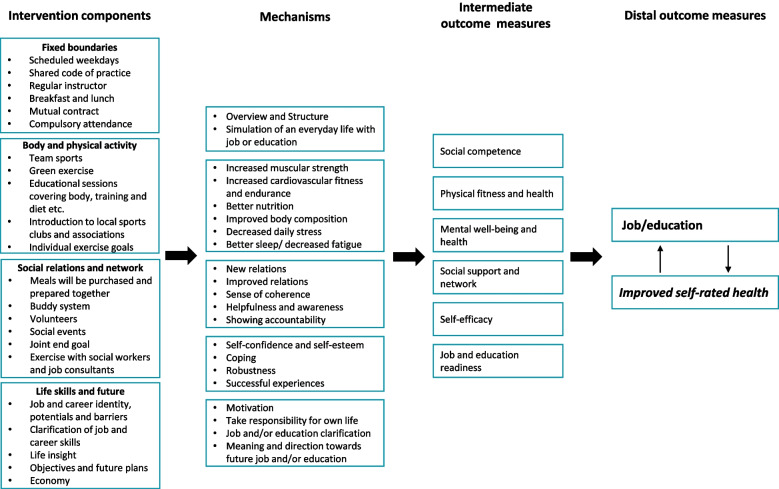
Program theory

The key findings emphasize the demand for new and innovative methods with a holistic approach to ensure a steady progression towards education or employment and that physical activities can be an evident element to include in future employment interventions for young people NEET. The NEXT STEP program theory or logic model according to intervention mapping [39] illustrates the four main components of the intervention based on the previously presented findings, the mechanisms, and the immediate and distal outcome measures (Fig. 1). In the design of the program theory, we used evidence from the Employability Indicator Project [9, 52–55] as well as other relevant literature [15, 19, 21, 42] describing mechanisms and intermediate outcome measures, hence leading to changes in our distal outcome measures: job/education and improved self-rated health.

The key ingredient in NEXT STEP is physical activity accompanied by three other components. In the following, we unfold the content of the intervention divided into the four overall components:

1. Fixed boundaries
2. Body and physical activity
3. Social relations and network
4. Life skills and future

Moreover, it is discussed how the elements of these components relate to the mechanism and outcomes in relation to progression towards education or employment.

#### Intervention component: fixed boundaries

The overall purpose of NEXT STEP fixed boundaries was to enhance attendance and motivation among young people NEET through a safe and recognizable setting. NEXT STEP fixed boundaries therefore include the following:

- Scheduled weekdays
- Shared code of practice
- Regular instructor
- Breakfast and lunch
- Mutual contract
- Compulsory attendance

With competent feedback from the practitioners in the municipalities, we decided to let the total duration be 16 weeks with mandatory attendance twice a week from 8:30 AM until 2:30 PM. Breakfast and lunch were included to ensure regularity as well as to increase attendance and motivation. The daily duration was chosen to simulate an ordinary weekday as if the participants were at work or at an educational institution, and the total duration of 16 weeks was recommended as a suitable period of time to get to know each other but also sustain the motivation among the instructors and young people NEET. The intervention should include fixed groups (not continuous recruitment) and a regular instructor to create a secure environment for the intervention. Additionally, a maximum of 15 participants were included in each group with the ideal number being around ten as recommended by the instructors. We thus expected a few young people NEET to withdraw from the intervention during the 16 weeks and considered the daily absence because of injuries, illness, enrollment in education, etc.

To ease the transition into NEXT STEP, we chose to include an easy start-up phase with days of shorter duration during the first few weeks when young people NEET are gradually introduced to the full program. To ease the group formation and to aid the feeling of community, we decided—as part of the start-up phase—to let young people NEET define a shared code of practice and to sign a mutual contract of commitment. The purpose of this component was to establish a foundation of accountability and to create a sense of coherence.

### Discussion of potential outcomes

All in all, it is known that employment entails latent psychosocial benefits such as time structure, common goals, and enforced activity [56, 57]. A period of unemployment, on the other hand, lacks these essential elements and thus is linked to poorer mental health and overall well-being [56, 58–61]. Goodman (2017) argues that for improvements in mental health, social leisure activities that provide a sense of time structure may be a low-cost intervention for unemployed [60], and Creed (2001) describes that community-based occupational training/personal development programs for unemployed can provide the latent functions that are missing for individuals without employment [62].

#### Intervention component: body and physical activity

The overall purpose of NEXT STEP body and physical activity was to introduce young people NEET to the beneficial physical, social, and mental effects of physical activity thereby possibly increasing the self-rated health of young people NEET. NEXT STEP body and physical activity includes the following:

- Team sports
- Green exercise
- Educational sessions covering the body, training, and diet, etc.
- Introduction to local sports clubs
- Individual exercise goals

There are consensus and consistent evidence that participation in team sports is associated with improvements in the psychological and social health compared to individual forms of physical activity [63]. According to a review by Andersen [64], team sports are beneficial for the psychosocial health because it offers participants an including, social, enjoyable, and meaningful setting with the possibility to develop psychosocial skills. Additionally, team sports, e.g., small-sided soccer games, are activities where participants report lower personally experienced strain compared to other types of exercise [65]. In team sports in general, numerous activity changes like accelerations, changes of directions, and jumps take place, and these are energetically very demanding and put a high cardiovascular load on the body [66]. Ultimate Frisbee has, besides the apparent physical element, an element of fair play, “Spirit of the Game,” where the participants themselves are referees, physical contact is not allowed, and after the game, each team will jointly evaluate how it went [67, 68]. NEXT STEP team sports include an introduction to basic team-based ball games and a gradual progression towards full games of Ultimate Frisbee.

Green exercise is a physical activity that takes place in outdoor natural environments or green spaces [69, 70]. Several studies have shown beneficial physical and psychological health effects of outdoor exercise [69, 71], and studies show additional positive effects of green exercise compared to training elsewhere/indoors [71, 72]. Moreover, green exercise can facilitate adherence to physical activity and minimize the perception of effort [73]. NEXT STEP green exercise includes activities with an inclusive and social focus (e.g., group challenges, physical games, and exercises in pairs), strength training with bodyweight, training on stairs and hills, and coordination and balance training with elements of parkour.

To sum it up, besides the benefits mentioned above, we thought that the simplicity of the green exercise and the possibility for individually adjusted exercises in combination with the necessity of dialog and cooperation in Ultimate Frisbee would be ideal for the target group in NEXT STEP. During the 16 weeks, participants will attend approx. 6 h of physical activity a week.

We were aware of the potential challenge of including participants with poor physical condition and limited knowledge of basic training in an intervention with physical activity. Therefore, we added a series of workshops to the intervention with a focus on basic nutrition, basic exercise physiology, sleep, and mental health to prepare young people NEET for the intervention ahead. We also included introductory visits to different local sports clubs and associations (a total of three throughout a project period). The primary purpose was to introduce young people NEET to other sports disciplines and to ensure sustainability by creating recognizability for the facilities and instructors at the specific sports clubs or associations, helping the transition out into the local sporting community. To increase motivation, we included individual exercise goals to visualize the physical progression throughout the intervention period.

### Discussion of potential outcomes

All in all, there is consensus that physical activity leads to physiological health benefits/improved physical fitness, including increased muscular strength and cardiovascular fitness [61]. Several psychological and social health benefits are associated with participation in physical activity with the most commonly mentioned being increased mental well-being and reduced stress and distress [62, 69–72]. Hult (2018, 2019) concludes that good physical fitness and health are important in maintaining good work ability during unemployment [74] and also argues that there is a need for intervention studies investigating the effects of increased exercise on health and work ability among long-term unemployed [75]. Moreover, according to Arendt (2016, 2017), there is a strong positive relationship between a better self-rated health and a chance of employment [9, 10]—the fewer health-related problems, the more time can be dedicated to job search and other related activities, etc. Thus, improved health and self-rated health are key indicators of progression towards employment [10, 53, 55].

#### Intervention component: social relations and network

The overall purpose of NEXT STEP social relations and network is to include young people NEET in a new community with positive social relations and support and to strengthen their social competencies. NEXT STEP social relations and network include the following:

- Meals purchased, prepared, and eaten together
- Buddy system
- Volunteers
- Social events
- Joint end goal
- Exercise with social workers and job consultants

As mentioned, and based on the exploratory study, we decided to include breakfast and lunch as a fixed part of the two NEXT STEP days. These meals will be purchased, prepared, and eaten together creating the setting of comfortable and informal talks where daily experiences and challenges can be debated. Moreover, to help young people NEET show up in the morning, we decided to include a buddy scheme where they either in pairs or small groups are responsible for each other’s attendance. The advantage of this scheme is that some young people will be assisted on the way with a phone call or a text message, and the more conscientious young people will benefit from taking responsibility helping the “buddy.”

NEXT STEP social relations and network also include a component with volunteers, who have several vital functions. Firstly, the volunteers will be practical assistance helping the instructors with the daily tasks making NEXT STEP possible, and secondly, the volunteers will be an objective third party set apart from both the municipality and the instructors. Thus, the idea is that young people NEET can share experiences with the volunteers and find comfort and social support talking with the volunteers if that is needed.

We also included social events and a common end goal to this intervention component. During the process, social events (e.g., a BBQ night) will visualize and strengthen the community. A final common goal (e.g., an adventure race) will both function as motivation during NEXT STEP and as a difficult however manageable hurdle to show young people NEET that they can manage tasks outside their comfort zone.

The exploratory study also revealed that the relationship between young people NEET and the instructor is essential, and, on this basis, the caseworker or another important contact person will be invited to join certain NEXT STEP activities throughout the period. The purpose is to strengthen their relationship and thus the cooperation when making the decision about the next employment measure or the most suitable education or employment for the young person NEET.

### Discussion of potential outcomes

With regard to social contact and employment, the Employability Indicator Project describes that both the ability to create and enter relationships and a network of professionals and friends/family are important factors in the progression towards employment or education [53, 55]. For the unemployed, social contact and social support during unemployment are positively associated with subjective well-being [56, 61], and maintaining personal relationships during a period of unemployment is associated with work ability [75]. For the unemployed, social activities that provide a sense of daily structure may be a promising approach to improving mental health [60].

#### Intervention component: life skills and future

The overall purpose of NEXT STEP life skills and future is to address individual challenges and to clarify the path towards education or employment. NEXT STEP life skills and future includes the following:

- Job and career identity, potentials, and barriers
- Clarification of job and career skills
- Life insight
- Objectives and future plans
- Economy

This component is delivered through motivating workshops. The workshops will focus on dialog and self-reflection instead of a more direct focus on education and employment. Education or employment is still the main goal, but as the key findings showed, young people NEET need personal and societal clarification before they can relate to realistic choices about their own future. To show consideration for the challenges related to school-based settings, NEXT STEP workshops will only last an hour including short breaks, and there will be a focus on using alternative teaching methods, e.g., educational videos and games.

Life skills and future will focus on self-reflection, job and educational identity, and clarification with regard to competencies, future possibilities, and personal barriers. In short, the participants will get insight into their current situation, discuss wishes and possibilities for the future, and through this process be guided and prepared to take responsibility for their life. Two primary methods have been chosen for future and competence: life psychology [76, 77] and future workshops [78].

Life psychology works with the persons as actors in their own life getting a handle on their own and shared existence [76, 77]. The purpose of this theory is to get a better grip on life: the fundamental task of establishing, sustaining, and improving both their own life and life together with others. A primary method and tool for dialog and self-reflection is the wheel of competence that assists the individual in reflecting on challenges and barriers moving towards a specific self-chosen goal in the near and distant future [76, 77].

Future workshops is an involving method where participants’ conception of their everyday life and ideas about the future are heard through brainstorms and dialog [78]. All the participants’ opinions are recognized, and in this case, statements from experts are not crucial. The method has previously been used among smokers in Danish vocational schools, and we see similarities with regard to the stigmatization that young people can experience [79]. The aim of this part of the workshops is to support the participants in articulating their own experiences and perspectives on the challenges of being NEET and afterwards have a shared brainstorming about common solutions before finally creating realistic solutions to the experienced challenges of being NEET. Additionally, we have included a specific element with a focus on the economy. The explanatory study showed that personal finances are a major area of concern. Two workshops during the 16 weeks are included to help the participants cope with their financial situation using alternative methods such as a finance game developed specifically for the group of young people [80].

.Finally, as previously described, there is a demand for an evaluation of the knowledge and competencies acquired and furthermore for a concrete short-term and long-term plan for the period after NEXT STEP. The instructors are responsible for having this dialog with each young person NEET during NEXT STEP and to inform the caseworker about the objectives and future plans.

### Discussion of potential outcomes

According to the Employability Indicator Project, job identity (meaning and direction) is a strong predictor of progression towards employment or education [9, 10, 55]. This indicator includes whether the individual has clear goals and wishes for the future but also whether there is a meaningful plan towards employment or education. Equivalently, Fugate (2004) also describes that career identity is central to a person’s employability. According to her, career identity is making sense of the past and present with the purpose of giving direction to the future [81]. In a review by Arendt & Jacobsen (2017), they conclude that guidance, upgrading of skills, and job training can have a positive influence on labor market readiness [10].

#### Intervention manual and training

A comprehensive intervention manual was prepared including background information and the theory of the different elements, a schedule for the full intervention, and daily programs described in detail with purpose, timetable, material preparation, etc. The manual is available on request (in Danish). Furthermore, instructors will receive the necessary upgrading of qualifications and training through a weekend course to ensure that the concept and theories are understood. Volunteers will participate in a full-day workshop that includes instructions in the program and what it means to be a volunteer for the target group, etc.

## Discussion and conclusion

Knowledge of effective employment interventions that can help young people NEET into education and employment is extensively demanded. Thus, different types of interventions are initiated to investigate how young people are best supported in starting and completing education or getting foothold in the labor market. At present, it is unclear whether the known effects are due to the specific interventions [1, 2] or rather other factors like general trends in society. Elements such as physical activity [1, 2] are included in many employment interventions, but there is great variation in the form and content of the interventions, and the effects on health and job-related outcomes have not been scientifically evaluated [3]. Therefore, knowledge of the effectiveness of specific elements focusing on health promotion and physical activity—both to promote health and to increase the likelihood of starting an education and or a job—is specifically demanded.

This paper presents experiences of using physical activity in employment intervention as well as the methods used for developing a health-promoting employment intervention with a focus on physical activity for young people NEET. The paper also includes the final main components of the NEXT STEP intervention: fixed boundaries, body and physical activity, social relations and network, and life skills and future. The NEXT STEP intervention was developed as an employment intervention equal to the already existing employment interventions in the Danish job centers. Eventually, a dialog between the young person NEET and the individual case worker may tell if an employment intervention with physical activity the right course of action for the young person NEET. The development was guided, though not rigorously, by intervention mapping, and the process of translating our key findings into intervention components took place in close cooperation with instructors and leaders working with young people NEET as well as other relevant project partners and researchers in the field.

Young people NEET is a very diverse target group, some of them struggling with a range of mental, physical, and social challenges. Some have had these challenges for years and maybe been part of the social system for years, whereas others have unexpectedly and suddenly ended up in a municipality-based employment intervention. This degree of complexity demands complex and holistic interventions helping young people NEET towards employment or education, and a few positive steps in the right direction may sometimes be accompanied by an equal number of steps in the opposite direction. The key findings from the exploratory study confirmed the importance of combining the health and employment effort for young people NEET to strengthen both the individual mental and physical health but also to ensure an ongoing progression towards education or employment. Overall, there is a demand for interventions that fully address the range of the physical, mental, and social barriers. A gradual and slow progression is of importance and must not be neglected.

Based on our findings, physical activities may be an essential element in employment interventions for young people NEET. However, the activities must be carefully planned and executed by competent and empathetic instructors to ensure balanced challenges that show consideration for individual social, physical, and mental barriers. Most importantly, mutual trust between young people NEET and the instructor as well as between young people NEET themselves must be established before a demand can be made on the individual contribution and effort. Moreover, as the findings also show, the physical activity cannot stand alone. So even though physical activity is the key ingredient of the intervention, other components focusing on other aspects (fixed boundaries, social relations and networks, life skills and future) are of great importance in order to ensure the progress towards a better state of health and education or employment.

The integration of both qualitative and quantitative data as well as the engagement with the setting in the development process was the most important strength of the current study. The intervention development process was initiated with the mixed-method mapping of Danish employment interventions with physical activity for young people NEET providing profound information on daily life in the Danish job centers. Additionally, we developed NEXT STEP in cooperation with experienced instructors working with young people NEET, job centers, and administrational leaders from different municipalities and other relevant researchers and partners. Therefore, decisions on the intervention design and components were carefully discussed with various stakeholders.

However, our study also has some limitations. First and foremost, we had a predefined aim and did not use a planning framework such as intervention mapping in a rigorous manner. In addition, we did not use behavior-oriented theories in the development of the intervention and its components as recommended in frameworks such as intervention mapping [39]. Even though the intervention was developed in cooperation with instructors, leaders, project partners, and researchers and through fieldwork in four municipalities, a broader needs and capacity assessment across different municipalities could have provided further information about the target group and the organizational context and capacities. In this regard, we could also have included the knowledge from general studies on interventions for young people NEET these may have been overlooked due to the predefined focus on physical activity.

Likewise, our close cooperation with instructors mostly keen on physical activities may have yielded insufficient perspectives on implementing employment interventions with physical activity among instructors of other opinions, thus hindering later dissemination and implementation. Participatory methodologies such as co-creation could also have strengthened the development, where young people NEET to a greater extent are involved in the decisions about the intervention design and specific intervention components. NEXT STEP has been developed solely in a Danish context, and the results of the development study may not necessarily be applicable to other countries.

## Conclusions

To the best of our knowledge, this is the first study investigating experiences of including physical activity in employment interventions to design a health-promoting employment intervention with a specific focus on physical activity for young people NEET, called NEXT STEP. The planning and development of the intervention indicate that a strong potential exists, and this has reinforced a pilot study before an actual implementation and evaluation of the effectiveness. These results will enhance the knowledge of how to promote NEXT STEP in the Danish municipalities and help young people NEET into education and employment*.*

## Supplementary Information

**Additional file 1.** Search string

**Additional file 2.** Overview of included articles

**Additional file 3: **GUIDED – a guideline for reporting for intervention development studies

## Data Availability

Data sharing is not applicable to this article as no datasets were generated or analyzed during the current study. Interview guides and transcripts used and/or analyzed during the current study are available from the corresponding author on reasonable request.

## Footnotes

1 Instructors are in this article used as an overall term for employees working with young people NEET in the Danish job centers such as caseworkers, vocational counselors, and similar terms

2 Væksthuset is a social enterprise and research center working to improve the employment effort for socially vulnerable groups of people in Denmark
