# Supplementary material for: The development of a health-promoting employment intervention with physical activity for young people Not in Education, Employment or Training (NEET): NEXT STEP—on the path to education and job
Source: Pilot Feasibility Stud. 2022 Oct 11;8:229. doi: 10.1186/s40814-022-01174-1 (PMC9551247; doi:10.1186/s40814-022-01174-1)
Supplement: Supplementary file 1 — Additional file 1. Search string [file 40814_2022_1174_MOESM1_ESM.pdf]

Additional file 1: search string

((((Physical activit*[Title/Abstract] OR physical-activit*[Title/Abstract] OR exercis*[Title/Abstract] OR sport*[Title/Abstract] OR fitness[Title/Abstract])) AND (un-employ*[Title/Abstract] OR unemploy*[Title/Abstract] OR re-employ*[Title/Abstract] OR reemploy*[Title/Abstract]))) AND (rtc[Title/Abstract] OR randomised[Title/Abstract] OR controlled[Title/Abstract] OR trial[Title/Abstract] OR intervention*[Title/Abstract]).
