# Supplementary material for: The development of a health-promoting employment intervention with physical activity for young people Not in Education, Employment or Training (NEET): NEXT STEP—on the path to education and job
Source: Pilot Feasibility Stud. 2022 Oct 11;8:229. doi: 10.1186/s40814-022-01174-1 (PMC9551247; doi:10.1186/s40814-022-01174-1)
Supplement: Supplementary file 2 — Additional file 2. Overview of included articles [file 40814_2022_1174_MOESM2_ESM.pdf]

Additional file 2: Overview of included articles

Author and year	Title	Design and participants	Intervention	Primary outcome	Results	Conclusion and perspectives
Kreuzfeld et al. 2013	Health effects and acceptance of a physical activity program for older long-term unemployed workers	Prospective pilot study with data collection at baseline, after intervention and six months after intervention, 119 long-term unemployed workers (53.7+- 3 years)	Three months low-threshold intervention: lectures for enhancing the individual health competence and a supervised physical training part in a fitness center	Nutritional status, cardiovascular risk factors, physical fitness, chronic backache and depression	Physical fitness improved significantly ($p<0.002$). Cardiovascular risk factors like systolic and diastolic blood pressure ($p<0.016$; $p<0.001$) and percentage body fat ($p<0.017$) decreased significantly and there was a reduction in depression ($p<0.028$)	The health promoting program reached people with an objective need to improve health. This may be the first step in regaining lost confidence and lowering the psychological barriers to employment placement.
Limm et al. 2015	Effects of a Health Promotion Program Based on a Train-the-Trainer Approach on Quality of Life and Mental Health of Long-Term Unemployed persons	Prospective parallel-group study, long-term unemployed, 287 participants (179 IG and 108 CG)	Three months, Individual sessions based on Motivational Interviewing and participatory group sessions involving physical activity	Health Related Quality of Life (HRQoL) (SF-12), depression and anxiety	HRQoL improved and anxiety and depression decreased significantly in the IG. A significant intervention effect was observed for anxiety ($p=0.012$). Effect sizes in the IG were small to moderate in terms of Cohen's d (anxiety: d=-0.33; SF-12 mental: d= 0.31; depression: d=-0.25; SF physical: d=0.19).	There is some evidence for an effectiveness of the intervention. They also indicate that a mix of individual and group interventions can be effective and improve the health-related quality of life and mental health of long-term unemployed persons. A participatory focus in the development of the program and continuous supervision of the health coaches may have contributes to the success of the program.
Schuring et al. 2009	Effectiveness of a health promotion programme for long-term unemployed subjects with health	RCT, 456 control group and 465 intervention group	Three sessions weekly over 12 weeks. One session a week was focused on education to	Perceived health and psychological measures (mastery, self-esteem and pain-	The intervention had no effect on mental and physical health, mastery, self-esteem and pain-related fear of movement nor on work	The intervention programme did not show beneficial effects. Integration into regular vocational

	problems: a randomized controlled trial		enhance the ability to cope with (health) problems, and two weekly sessions consisted of physical activities.	related fear of movement). Secondary outcome measures were work values, job search activities and reemployment	values, job search activities and re-employment	rehabilitation activities may have negatively affected the results of the programme.
Schutgens et al. 2009	Changes in physical health among participants in a multidisciplinary health programme for long-term unemployed persons	Longitudinal, non-controlled design: 252 persons had complete data collection at baseline	Two weekly exercise sessions and one weekly cognitive session during 12 weeks	BMI, blood pressure, cardiorespiratory fitness, abdominal muscle strength, and low back and hamstring flexibility	Participant's cardiorespiratory fitness, abdominal muscle strength and flexibility had increased whereas blood pressures had decreased. Effect sizes ranging from 0.17-0.68.	Physical health improved after participation and participants with the poorest physical health benefited most from the programme
Watson et al. 2003	Returning the chronically unemployed with low back pain to employment	Pre-post study, 86 long-term unemployed people	Six weeks including 12 half days with additional vocational counselling, occupationally-oriented rehabilitation programme using cognitive behavioral training supported by physical activity	Return to work and progress towards work	At follow-up 38.4% of subjects were employed and another 23% were in voluntary work or education/training. Those who failed to make positive progress were characterized by longer period of unemployment and higher scores on somatic anxiety and depression.	A combined approach integrating health and vocational approaches may be effective in returning people to employment or other productive activity.
